# Neonatal *Moraxella osloensis* Ophthalmia

**DOI:** 10.3201/eid1111.050488

**Published:** 2005-11

**Authors:** Andrew Walls, Ellen Wald

**Affiliations:** * University of Pittsburgh Medical Center, Pittsburgh, Pennsylvania, USA; † University of Pittsburgh School of Medicine, Pittsburgh, Pennsylvania, USA

**Keywords:** Moraxella, Ophthalmia neonatorum, Conjunctivitis, letter

**To the Editor:** *Moraxella osloensis* is an aerobic, gram-negative, lactose-nonfermenting coccobacillus. It is a commensal of the human upper respiratory tract and occasionally of the skin and urogenital tract (*1*). Unlike *M. catarrhalis*, *M. osloensis* is rarely pathogenic in humans. However, several cases of serious infections caused by this organism have been documented (*2**–**6*). While cases of nongonococcal, nonchlamydial, neonatal ophthalmia have been reported in which the causative agent was *M. catarrhalis* (*7**,**8*), to our knowledge, this case is the first report of neonatal ophthalmia due to *M. osloensis*.

A 3-week-old, previously healthy boy was seen at the emergency department with a 48-hour history of yellow drainage from and swelling in both eyes. One day before admission, the drainage increased; the child could not open his eyes spontaneously. He had been eating well and was normally active. Aside from mild fussiness, no other symptoms were noted.

The child was born full-term without complications to a gravida 6, para 5–6 mother. He received normal newborn care, including topical erythromycin ointment to the eyes. Aside from some mild jaundice at 6 days of age, he had been healthy. The mother denied any history of sexually transmitted disease.

On examination, the infant's temperature was 38°C rectally, heart rate 144 beats/min, respirations 26/min, and blood pressure 94/60 mm Hg. The child appeared well developed and was fussy but showed no symptoms of toxicity. Both eyelids were markedly swollen and erythematous, and a yellow, purulent discharge was noted bilaterally. The sclera and conjunctivae were injected bilaterally. An ophthalmologist recorded that the red reflex was intact bilaterally and the corneas were clear. Intravenous cefotaxime, oral erythromycin, and topical erythromycin ointment to the eyes were recommended. The leukocyte count was 11,400 cells/mm^3^ with a normal differential. Hemoglobin level, hematocrit, platelet count, and bilirubin level were all within normal range. Urinalysis results as well as urine, blood, and cerebrospinal fluid cultures were negative. Secretions from the eyes were collected and sent for Gram stain and bacterial culture as well as chlamydial culture. Gram stain showed few gram-variable cocci.

The child's eyes were flushed with copious amounts of normal saline, and a dose of intravenous cefotaxime and ampicillin was administered in the emergency department. He was admitted to the hospital for presumed ophthalmia neonatorum. The following day, decreased lid swelling, erythema, and eye discharge were observed, with trace conjunctival injection and minimal chemosis.

The child's condition improved markedly during the next 24–48 hours. Cultures of the secretions obtained from the eye grew presumed *Neisseria* species as a pure culture. The isolate was sent to the Allegheny County Health Department for further testing and speciation. It was first tested with a fluorescein-conjugated antibody for *Neisseria gonorrhoeae*; results were negative. A RapID NH panel (Remel, Lenexa, KS, USA) was performed that identified the isolate as *M. osloensis* with a 99.7% probability. Ideally, the isolate would have undergone more comprehensive genotypic and phenotypic characterization. However, as a presumed *Neisseria* species, it was subjected to the usual testing protocol at the health department. Chlamydial culture was performed by using buffalo green monkey kidney cells (Viromed, Minnetonka, MN, USA) grown under standard conditions. No viral inclusions were seen, and the culture did not react with chlamydial antibodies (Trinity Biotech, Bray, Ireland). Because the child responded rapidly to antimicrobial drug treatment, no further workup of the bacterial isolate was considered. The child was healthy 3 days later and was discharged to his home with topical erythromycin and instructions to his parents to follow up with his primary care physician.

Neonatal ophthalmia is a potentially serious, sight-threatening infection that may be caused by sexually transmitted pathogens. Accordingly, this clinical presentation warrants prompt diagnosis and appropriate therapy. At the same time, suspicion of a sexually transmitted disease causes immense social turmoil. Specific bacterial cultures are essential for precise microbiologic diagnosis and treatment.

Cultures of conjunctival specimens from our patient grew *M. osloensis*. Clinically, this patient's infection was indistinguishable from other causes of neonatal ophthalmia. The differential diagnosis includes other agents such as *N. gonorrhoeae*, *Chlamydia trachomatis*, *M. catarrhalis*, *Staphylococcus aureus*, *Haemophilus influenzae*, and *Streptococcus pneumoniae*. Rarely, gram-negative enteric organisms may be implicated (*9*). Viruses, such as adenovirus or herpesvirus, are also a potential cause but were unlikely in this case.

Finally, social issues must be considered. When an infant is seen with neonatal ophthalmia, a physician will often presume it to be gonococcal or chlamydial and assume the mother is positive for these infections. Recognizing that *Moraxella* species, including *M. osloensis*, may produce an identical clinical picture should limit presumptions regarding sexually transmitted diseases until a precise microbiologic diagnosis is made.
